# Cost-Effectiveness of Capsaicin 8% Patch Compared with Pregabalin for the Treatment of Patients with Peripheral Neuropathic Pain in Scotland

**DOI:** 10.1371/journal.pone.0150973

**Published:** 2016-03-16

**Authors:** Colette Mankowski, Sachin Patel, David Trueman, Anthony Bentley, Chris Poole

**Affiliations:** 1 Astellas Pharma Europe Ltd., Chertsey, Surrey, United Kingdom; 2 Astellas Pharma UK Ltd, Chertsey, Surrey, United Kingdom; 3 Abacus International, Bicester, Oxfordshire, United Kingdom; Harvard Medical School, UNITED STATES

## Abstract

We evaluated the cost-effectiveness of capsaicin 8% patch (QUTENZA™) versus pregabalin in patients with PNP from the perspective of the National Health Service (NHS) and Personal and Social Services in Scotland, UK. A decision-tree cost-effectiveness model was developed for non-diabetic patients with peripheral neuropathic pain (PNP) who were pregabalin-naïve and had not achieved adequate pain relief or tolerated conventional first- or second-line treatments. Patients entering the model received either a single application of capsaicin 8% patch or titrated daily dosing with pregabalin; after 8 weeks patients were classified as responders, non-responders, or were assumed to discontinue treatment due to intolerable adverse events. Responders continued to receive baseline treatment at intervals observed in clinical practice. Non-responders and those who discontinued treatment were assumed to receive last-line therapy (duloxetine). The base-case time horizon was 2 years. Model inputs for effectiveness, discontinuations and health-state utilities were taken from a head-to-head non-inferiority study (ELEVATE, NCT01713426). Other inputs were obtained from published sources or clinical expert opinion. Costs were expressed in GBP 2013/14. Results were presented as incremental cost-effectiveness ratios (ICER), i.e. cost per quality-adjusted life-year (QALY) gained. Model assumptions were tested with scenario analyses. Parameter uncertainty was tested using one-way and probabilistic sensitivity analyses. Compared with dose-optimized pregabalin, capsaicin 8% patch was the dominant treatment strategy (total cost difference, –£11; total QALY gain, 0.049). Capsaicin 8% patch was also the dominant treatment strategy versus pregabalin in 6 out of 7 scenario analyses. The model was most sensitive to variation in time to capsaicin 8% patch retreatment (maximum ICER, £7,951/QALY at lower-bound 95% confidence interval). At a willingness-to-pay threshold of £20,000/QALY, the probability of capsaicin 8% patch being cost-effective versus pregabalin was 97%. Capsaicin 8% patch is a cost-effective treatment option compared with dose-optimized pregabalin in patients with PNP who have failed one or more previous systemic treatments.

## Introduction

Neuropathic pain is a clinical description, rather than a diagnosis, and is defined as pain caused by a lesion or disease of the central or peripheral somatosensory nervous system [1]. It is a common debilitating condition; epidemiological studies performed in the UK and France suggest that 6.9% to 8.2% of the general population experience pain with neuropathic characteristics [2, 3]. Patients with neuropathic pain report significantly greater impairment of health-related quality of life (HRQoL), more sleep problems, and worse anxiety and depression scores than individuals with non-neuropathic pain [4]. Patients also use significantly more healthcare resources (including physician visits, specialist visits, and drug treatments for pain) than those with non-neuropathic pain or no pain [4].

Managing patients with chronic neuropathic pain is challenging, because many patients obtain incomplete pain relief or experience intolerable and/or dose-limiting adverse effects to drug treatment [5]. The Scottish Intercollegiate Guidelines Network (SIGN) recommend oral drugs, specifically amitriptyline or gabapentin, for the initial treatment of neuropathic pain [6]. Pregabalin is recommended when other first- and second-line treatments have failed [6].

Capsaicin 8% patch is licensed in Europe for the treatment of peripheral neuropathic pain (PNP) in adults, either alone or in combination with other therapies [7]. Clinical trials show that capsaicin 8% patch provides effective pain relief in patients with post-herpetic neuralgia [8, 9] and HIV-associated neuropathy [10]. More recently, a large randomized head-to-head study (ELEVATE) showed that capsaicin 8% patch was non-inferior to pregabalin for pain reduction after 8 weeks in a population of non-diabetic patients with PNP of mixed aetiologies [11]. Capsaicin 8% patch is generally well tolerated, and the most common adverse events are transient mild-to-moderate application site reactions, such as pain and erythema [8–10].

We report the findings from a cost-effectiveness analysis which compared capsaicin 8% patch with dose-optimized pregabalin in non-diabetic patients with PNP from the perspective of the National Health Service (NHS) and Personal and Social Services in Scotland.

## Methods

### Model overview

**Fig 1:**
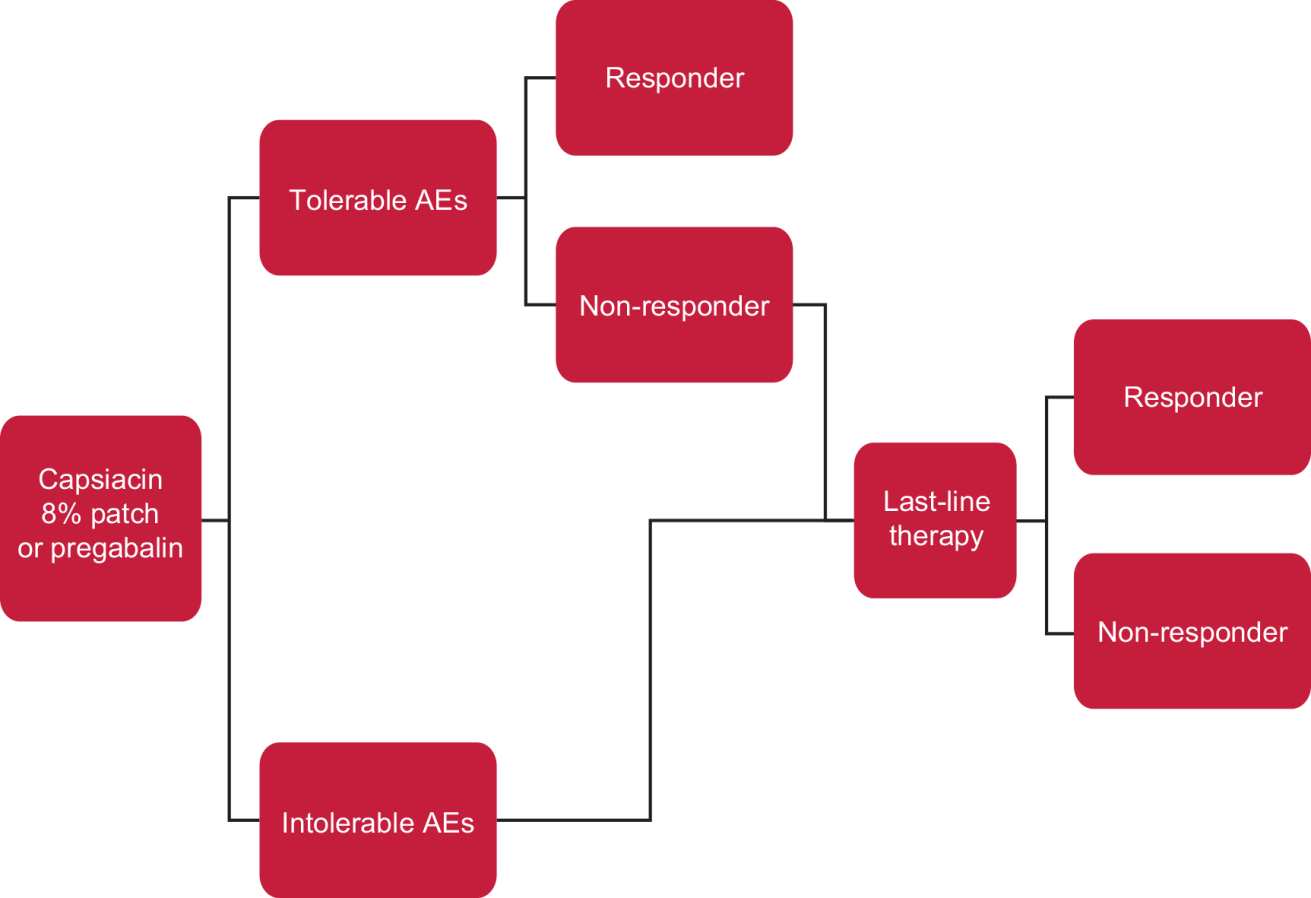
Decision-tree model. **Key:** Responder: ≥30% reduction in “average pain for the past 24 hours” according to the NPRS score. Non-responder: <30% reduction in “average pain for the past 24 hours” according to the NPRS score. Tolerable AE: persistent AE typically including dizziness, nausea and somnolence, which do not result in treatment discontinuation. Intolerable AE; AE that result in treatment discontinuation. *Abbreviations*: AE, adverse event; NPRS, Numeric Pain Rating Scale.

A cost-utility model using a decision-tree approach was developed in Microsoft^®^ Excel 2010 to predict the cost-effectiveness of capsaicin 8% patch versus pregabalin in non-diabetic patients with PNP for whom conventional first- and second-line treatments have been ineffective or not tolerated (Fig 1). The model was conducted from the perspective of the NHS and Personal and Social Services in Scotland, UK. The model had a base-case time horizon of 2 years, based on clinical expert advice that patients with PNP would unlikely remain on any prescribed treatment, including capsaicin 8% patch, for >2 years. The UK clinical expert panel included pain physicians (n = 3), pain nurses (n = 4), a senior academic (n = 1); and a general practitioner [GP] (n = 1). Other time horizons, up to 10 years, were modelled in scenario analyses. Costs and outcomes were assigned during the first year of treatment; these were then extrapolated beyond the first year by applying constant health-state costs and health-state utilities, both discounted at 3.5% [12].

Patients entered the model and received either capsaicin 8% patch or pregabalin (Fig 1). It was assumed that patients either remained on therapy or discontinued treatment due to intolerable adverse events. Patients who remained on therapy were classified as responders or non-responders after 8 weeks, corresponding to either a ≥30% or <30% decrease, respectively, in “average pain for the past 24 hours” on the numeric pain rating scale (NPRS) from baseline. A 30% threshold reduction in pain was selected to define responders as it is consistent with SIGN guidelines [6] and consistent with a clinically meaningful improvement in chronic pain [13]. The 8-week assessment period to determine response status is consistent with the duration of treatment in the ELEVATE study [11], and is the recommended time to stop treatment if sufficient benefit is not observed [14]. Responders continued to receive their baseline treatment and were assumed to respond for the remainder of the analysis. Non-responders and patients who discontinued due to intolerable adverse events were assumed to receive ‘last-line’ therapy. In this model, duloxetine was chosen as ‘last-line’ therapy as a proxy for the various therapies that may be offered at this stage of the treatment pathway.

Input data for clinical effectiveness, discontinuation rates and health-state utilities were based on the ELEVATE study and are described in detail below. Other model inputs were obtained from the published literature or expert UK clinical opinion. Three members of the panel practise in Scotland and the remaining six in England and Wales. Further advice regarding assumptions made in the model was also sought from a pain specialist practising in Scotland.

### Patient population

The model considered non-diabetic patients with PNP who were pregabalin-naïve (or who had not received adequate treatment with pregabalin according to investigator opinion) and who had not achieved adequate pain relief from or had not tolerated conventional first- and second-line treatments (amitriptyline or gabapentin [6]). This population is consistent with the recommended use of the capsaicin 8% patch by NHS Scotland [14].

### Treatments

Two treatments were considered in the model: (i) capsaicin 8% cutaneous patch; and (ii) oral pregabalin 150 mg/day (i.e. starting dose of two 75 mg capsules) titrated to an optimized dose (maximum 600 mg/day) over 10 to 14 days aligned with its indication [15]. Pregabalin was selected as the primary comparator as it is typically used in Scotland when first- and second-line pharmacological treatments have failed [6].

Last-line therapy, defined by clinical expert panel opinion, was given to patients who had failed to achieve an adequate response with capsaicin 8% patch or pregabalin in the secondary care setting. At the stage of last-line therapy, no single treatment pathway predominates in Scotland and it may consist of many treatments including opioids, other pharmacologic interventions, and multidisciplinary assessment with psychological therapy [6]. For simplicity, duloxetine 60 mg/day was selected as a reasonable proxy for last-line therapy [6], and was used to provide a conservative estimate of the cost and benefits of such treatments.

### Model input parameters

Summaries of model inputs are presented in Tables 1 and 2, and model assumptions are listed in S1 Table.

**Table 1:** Model inputs: efficacy and utilities.

Parameter	Base-case value	Sensitivity analysis values	Source
**Efficacy**			
Probability of response, %			
Capsaicin 8% patch	55.7	49.9–61.5	Haanpää et al. 2016 [11]
Pregabalin	54.5^a^	48.7–60.4	Haanpää et al. 2016 [11]
Last-line therapy	20.0	15.0–45.0	Clinical expert estimate
Discontinuation due to adverse events, %			
Capsaicin 8% patch	0.0	0.0–0.0	Haanpää et al. 2016 [11]
Pregabalin	8.5	5.2–11.8	Haanpää et al. 2016 [11]
Time to onset of response,^b^ days			
Capsaicin 8% patch	7.5	6.0–10.0	Haanpää et al. 2016 [11]
Pregabalin	36.0	22.0–50.0	Haanpää et al. 2016 [11]
Time to capsaicin retreatment, days	179	117–241	Poole et al. 2013 [16]
**Utilities**			
Baseline/no response	0.57	0.55–0.58	Astellas, data on file [17]
Response with capsaicin 8% patch	+0.23^c^	0.20–0.26	Astellas, data on file [17]
Response with pregabalin	+0.20^c^	0.17–0.23	Astellas, data on file [17]
Response with last-line therapy	+0.23^c^	0.20–0.26	Assumption

^a^As ELEVATE demonstrated non-inferiority of capsaicin 8% patch compared with pregabalin in the control of pain, it was assumed that both treatments were equivalent in the base-case analysis (using the capsaicin response rate). A scenario analysis using the actual reported efficacy was also conducted.

^b^Number of days when 50% of patients showed a response over 3 consecutive days.

^c^Change from baseline.

**Table 2:** Model inputs: costs.

Parameter	Base-case value	Sensitivity analysis values	Source
**Costs**			
Capsaicin 8% patch			
Acquisition cost per patch	£210	–	BNF 2014 [18]
Mean no. patches/treatment	1.38^a^	1.26–1.51	Haanpää et al. 2016 [11]
Nurse time	£59.50^b^	£29.75–£119	Curtis 2013 [19]; nurse opinion
Pair of nitrile gloves	£0.06	£0.05–£0.08	CCS Direct 2014 [20]
*Total per treatment*	£349.99		
Optional topical anesthesia^c^			
Lidocaine 4% acquisition cost per treatment	£30.91	–	BNF 2014 [18]
Tegaderm^®^ film acquisition cost per treatment	£3.28	–	BNF 2014 [18]
*Total per treatment*	£34.19		BNF 2014 [18]
Pregabalin			
Acquisition cost per tablet	£1.15	–	BNF 2014 [18]
*Total per annum*	£839.50^d^		
Last-line therapy			
Duloxetine acquisition cost per tablet^e^	£0.99	£0.74–£1.24	BNF 2014 [18]
*Total per annum*	£361.35		
Intolerable adverse events			
GP consultation	£45^f^	£34–£66	Curtis 2013 [19]
Pain specialist follow-up visit	£125	£90–171	Department of Health 2013/14 [21]
**Discount rates**			
Costs, %	3.5	1.5–6	SMC 2014b [12]
Utilities, %	3.5	1.5–6	SMC 2014b [12]

^a^Total acquisition cost of £290.43 per treatment based on 1.38 patches per treatment.

^b^Band 6 nurse at £119 per hour, assuming 30 minutes of patient contact time. For the sensitivity analyses, the contact time was varied from 15 to 60 minutes.

^c^Included in sensitivity analysis only. See text for application rates and assumptions.

^d^Assuming that patients received a twice-daily regimen.

^e^Duloxetine (60 mg/day starting dose, up to a maximum of 120 mg/day) [22] was used as a proxy to estimate the cost of last-line therapy.

^f^Contact lasting 11.7 minutes.

*Abbreviation*: GP, general practitioner; BNF, British National Formulary; CCS, Castle Cleaning and Safety; SMC, Scottish Medicines Consortium.

### Efficacy

The probabilities of a response at 8 weeks, discontinuation due to intolerable adverse events and time to onset of pain relief for capsaicin 8% patch and pregabalin were taken from the ELEVATE study [11, 17]. The probability of a response at 8 weeks with last-line therapy was based on clinical expert opinion. For the base-case analysis, it was assumed that patients who responded continued to do so for the remainder of the analysis.

### Health-state utilities

Health-state utility scores were applied for the duration of the model and were derived from EQ-5D-5L scores reported in the ELEVATE study (Table 1) [17]. UK-specific tariffs for EQ-5D-5L were derived from mapping between EQ-5D-5L and EQ-5D-3L scores [23], as country-specific tariffs for EQ-5D-5L were not available at the time of analysis. No Scottish tariff for either form of the EQ-5D index currently exists.

At model entry, patients were assumed to experience HRQoL levels observed at baseline in the ELEVATE study. Health benefits within the model were based on HRQoL improvements resulting from the successful treatment of baseline pain. For simplicity, the model assumed that patients who responded to treatment experienced a linear increase in utility from the baseline value by the increment associated with pain relief for the median time to pain relief (S2 Fig). Responders continued to respond to that treatment for the period modelled and were assigned the relevant utility score for their pain relief. It was conservatively assumed that patients receiving last-line therapy would respond immediately, and that the health benefit was equal to that the utility score improvement for capsaicin 8% patch.

### Resource use and costs

Costs were evaluated from a NHS Scotland and Personal and Social Services payer perspective. For the base-case analysis, these included drug acquisition costs, patch application costs (i.e. nurse time and sterile gloves), and GP and pain specialist visits (Table 2).

In the base-case analysis, patients incurred the annual cost of the treatment they responded to (i.e. capsaicin 8% patch, pregabalin or last-line therapy). Patients who discontinued treatment and became eligible for last-line therapy were assumed to be seen by a GP and then referred to a pain specialist. The costs of routine monitoring were not included, as monitoring was assumed to occur at equal frequencies with both treatment strategies. Costs associated with adverse events not leading to treatment discontinuation were also conservatively not included. Patients receiving last-line therapy were assumed to continue to incur the cost of therapy regardless of response status.

The cost of treatment with the capsaicin 8% patch was based on the average number of treatments in a 1-year period. The mean number of capsaicin patches used per treatment was 1.38 (standard deviation [SD] 1.08), taken from the ELEVATE study [11]. Mean data are preferable to median data in health economic modelling [24] because the total cost of care can be calculated from mean cost per patient, but not from the median as it’s affected by positively skewed data [25]. Thereby, the mean time to re-treatment for patients responding to their first capsaicin patch (179 days, 95% CI 117–241) was taken from an interim analysis of ASCEND, a European non-interventional study on the use of capsaicin 8% patch in standard clinical practice [16]. It was assumed that one pair of nitrile gloves and 30 minutes’ nurse contact time were required for each patch application, based on expert clinical opinion and the Unit Costs of Health and Social Care [19].

Recent evidence has demonstrated that there is no statistically significant difference in clinically meaningful treatment-related discomfort between patients treated with capsaicin 8% patch who received pretreatment with a topical anesthetic and those who did not receive pretreatment [26]. As a consequence, the license for capsaicin 8% patch has been updated to reflect the fact that topical anesthesia prior to application is optional [7]. Therefore, pre-application topical anesthesia was not included in the base-case analysis, but was considered in a scenario analysis as some patients may require topical anesthesia [7].

To estimate the annual cost of pregabalin, it was assumed that patients received a twice-daily dosing regimen, as pregabalin tablets of all strengths are identically priced (i.e. £1.15 per tablet). The cost of duloxetine 60 mg once daily was used as a proxy for the cost of last-line therapy. It was assumed that adherence with pregabalin and last-line therapy was 100%.

Costs were based on 2013/2014 British Pounds. Costs and outcomes were discounted at 3.5% after the first year in line with advice from the Scottish Medicines Consortium [12].

### Model outputs

The results were presented as incremental cost-effectiveness ratios (ICERs), i.e. cost per quality-adjusted life-year (QALY) gained. Total costs and QALYs for each treatment strategy were reported as secondary outcome measures.

### Scenario and sensitivity analyses

A scenario analysis in which pain relief with capsaicin 8% patch decreased over time between retreatments was conducted. The ELEVATE study showed that capsaicin 8% patch reduced pain over an 8-week period [11], and a post-hoc analysis suggested that approximately 39% of its treatment effect, with regards to HRQoL, was lost after the 8-week period in patients requiring re-treatment [17]. Therefore, in this scenario, it was assumed that pain relief started to decrease by 39% after 8 weeks until the time of re-treatment, but was restored to the level of the original response within two weeks of re-treatment (S1 Fig).

A scenario analysis for pre-application of topical anesthesia prior to capsaicin 8% patch was also conducted. For this scenario, lidocaine 4% cream (LMX 4^®^) was assumed to be applied at a rate of 45 mg per patch, followed by application of an occlusive film (Tegaderm^®^). Assuming 1.38 patches per treatment, the estimated per treatment cost of lidocaine cream and occlusive film was £30.91 and £3.28, respectively [18]. As an alternative to topical anesthesia, oral tramadol 50 mg may also be used prior to application of capsaicin 8% patch [7]. However, this option was not considered as a scenario analysis as it is considerably less costly (approximately £0.04 per 50 mg tablet [18]) than topical anesthesia.

A structural sensitivity analysis was conducted to determine the impact of varying the time horizon between 1 and 10 years. Deterministic sensitivity analyses to test parameter uncertainty were also performed, where all model parameters were systematically and independently varied over plausible ranges determined either by the 95% confidence intervals [27] or, in the absence of a reported confidence interval, an assumed variation of ±25% of the point estimate [28]. ICERs were estimated for the upper and lower values, and presented in a tornado diagram. Threshold analyses were conducted on the 10 parameters that had the greatest impact on the ICER. Willingness-to-pay threshold limits of £20,000 and £30,000 per QALY gained were applied, to be aligned with guidance reported by the Scottish Medicines Consortium and NICE [12, 29]. Joint parameter uncertainty was explored through probabilistic sensitivity analysis, whereby all parameters were assigned distributions and varied simultaneously. Ten thousand Monte Carlo simulations were performed and results were presented as a cost-effectiveness plane and a cost-effectiveness acceptability curve.

## Results

### Base-case analysis

The results of the base-case analysis over a 2-year time horizon are presented in Table 3. Compared with pregabalin, the capsaicin 8% patch treatment strategy was dominant (more effective with lower costs) with a total cost saving of £11 and QALY gains of 0.049.

**Table 3:** Base-case analysis (2-year time horizon).

	Capsaicin 8% patch	Pregabalin	Capsaicin 8% patch vs pregabalin^a^
**Mean costs per patient treated**			
Capsaicin 8% patch	£915	–	£915
Pregabalin^b^	–	£881	–£881
Last-line therapy	£282	£312	–£30
GP/pain specialist visits	£0	£14	–£14
*Total*	£1,197	£1,207	–£11
**Mean QALYs per patient treated**			
*Total*	1.360	1.310	0.049
**ICER**			**Dominant**

^a^Values subject to rounding.

^b^Daily optimized dose.

*Abbreviations*: ICER, incremental cost-effectiveness ratio; QALY, quality-adjusted life year.

### Sensitivity analyses

#### Scenario analyses

Capsaicin 8% patch was the dominant treatment strategy versus pregabalin in six of the seven scenario analyses (Table 4). Only the inclusion of optional topical anesthesia prior to treatment with the capsaicin 8% patch resulted in an incremental cost, but the treatment strategy remained cost-effective (ICER £1,599 per QALY).

**Table 4:** Scenario analyses.

Scenario		Capsaicin 8% patch	Pregabalin	Capsaicin 8% patch vs pregabalin^a^
Difference in clinical efficacy (capsaicin 8% patch: 55.67%, pregabalin: 54.51%)	Total costs	£1,197	£1,198	–£2
	Total QALYs	1.360	1.307	0.052
	ICER	**Dominant**		
No difference in time to response (set time to response to 7.5 days)	Total costs	£1,197	£1,207	–£11
	Total QALYs	1.360	1.314	0.045
	**ICER**	** Dominant**		
No difference in discontinuation due to intolerable adverse events (set rate of discontinuations with pregabalin to 0%)	Total costs	£1,197	£1,233	–£36
	Total QALYs	1.360	1.324	0.036
	**ICER**	** Dominant**		
No difference in pain response utilities (set utility associated with pregabalin response to 0.23)	Total costs	£1,197	£1,207	–£11
	Total QALYs	1.360	1.338	0.021
	**ICER**	** Dominant**		
No difference in clinical efficacy, time to response, discontinuation due to intolerable adverse events or pain response utilities	Total costs	£1,197	£1,233	–£36
	Total QALYs	1.360	1.360	0.0000
	**ICER**	** Dominant**		
Patients experience a decrease in perceived pain relief over time with capsaicin 8% patch before subsequent retreatments	Total costs	£1,197	£1,207	–£11
	Total QALYs	1.327	1.310	0.017
	**ICER**	**Dominant**		
Base-case assumptions but with the inclusion of topical anesthesia prior to capsaicin 8% patch treatment	Total costs	£1,286	£1,207	£79
	Total QALYs	1.360	1.310	0.049
	**ICER**			**£1,599**

^a^Values subject to rounding error.

*Abbreviations*: ICER, incremental cost-effectiveness ratio; QALY, quality-adjusted life year.

### Structural analysis

Using a 1-year time horizon, the ICER for capsaicin 8% patch versus pregabalin increased to £1,242 per QALY. For all other time horizons (i.e. 2 to 10 years), capsaicin 8% patch was the dominant treatment strategy.

### One-way sensitivity analysis

**Fig 2:**
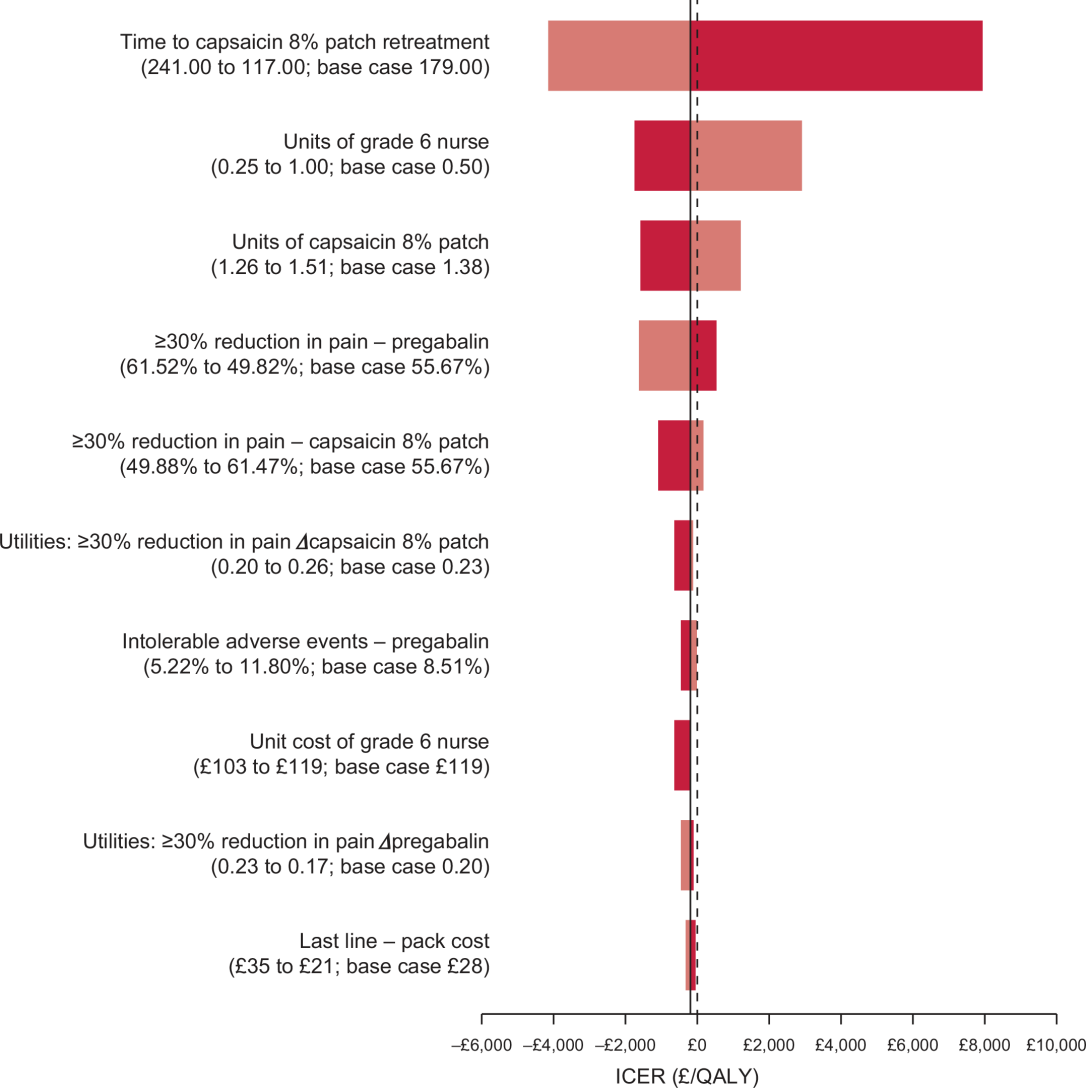
One-way sensitivity analysis (2-year time horizon). **Key:** Each bar represents the ICER values associated with using the low (dark shading) and high (light shading) parameter values. The dotted line represents the base-case ICER.

The 10 variables that had the greatest impact on the ICER are presented in Fig 2. The capsaicin 8% patch was dominant or cost-effective (less than a willingness-to-pay threshold of £20,000 per QALY) at all values tested. The ICER was most sensitive to variations in the time to re-treatment with the capsaicin 8% patch. At the low value (117 days), the ICER increased to £7,951 per QALY, whereas at the high value (241 days), the capsaicin 8% patch was the dominant treatment strategy. Other variables for which capsaicin 8% patch was cost-effective rather than dominant were: grade 6 nurse time (high value ICER £2,941); number of capsaicin 8% patches per treatment (high value ICER £1,188); proportion of responders with pregabalin (low value ICER £532) and capsaicin 8% patch (high value ICER £164).

The threshold analysis showed that in most cases, a value to yield an ICER of £20,000 or £30,000 per QALY could not be determined or was outside a plausible range (Table 5). Capsaicin 8% patch only failed to be cost-effective at a £30,000 per QALY willingness-to-pay threshold when the time to re-treatment was set at less than 60.46 days, or the average number of patches per treatment exceeded 4.10. If the cost of pregabalin was set at £0, the ICER for capsaicin 8% patch versus pregabalin was £17,650 per QALY.

**Table 5:** Threshold analysis.

Variable	Base case	£20,000/ QALY	£30,000/ QALY
Time to capsaicin 8% patch retreatment, days	179.00	77.43	60.46
Grade 6 nurse time, hours	0.50	3.70	5.29
Number of capsaicin 8% patches per treatment	1.38	3.20	4.10
Responders^a^ with pregabalin, % of patients	55.67	NA	NA
Responders^a^ with capsaicin 8% patch, % of patients	55.67	NA	NA
Utilities for response with capsaicin 8% patch, change from baseline	0.2284	NA	NA
Intolerable adverse events with pregabalin, % of patients	8.51	NA	NA
Unit cost of grade 6 nurse, £ per hour	119	882	1,259
Utilities for response with pregabalin, change from baseline	0.1989	NA	NA
Cost per pack of last-line therapy, £	27.72	NA	NA

^a^Defined as ≥30% decrease in “average pain for the past 24 hours” numeric pain rating scale score from baseline.

*Abbreviations*: NA, not applicable (i.e. target ICER could not be achieved with a plausible value); QALY, quality-adjusted life year.

### Probabilistic sensitivity analyses

**Fig 3:**
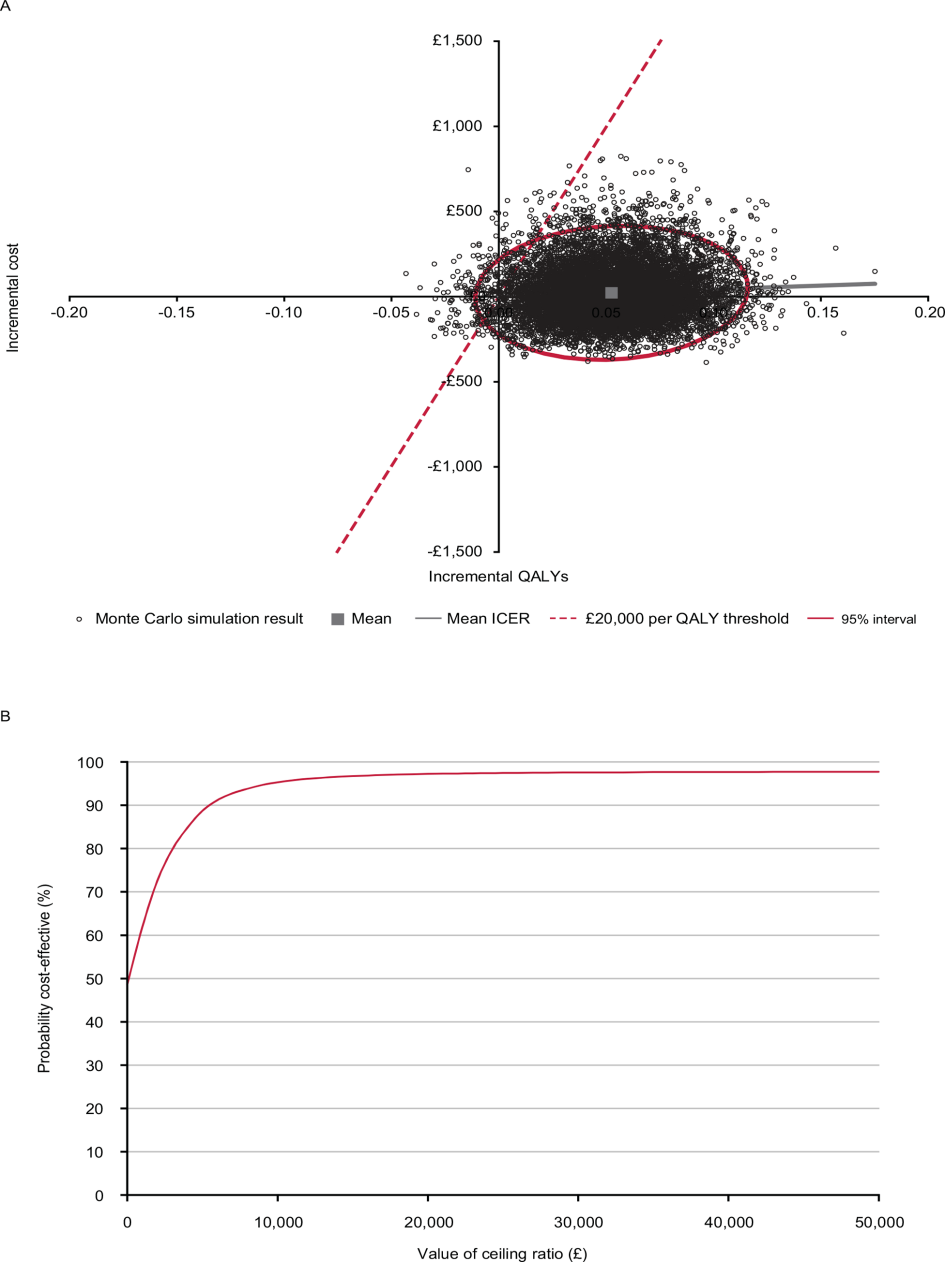
**Probabilistic sensitivity analysis (2-year time horizon): (A) cost-effectiveness plane and (B) cost-effectiveness acceptability curve for capsaicin 8% patch versus pregabalin.** *Abbreviations*: ICER, incremental cost-effectiveness ratio; QALY, quality-adjusted life year.

The results from the probabilistic sensitivity analysis are presented in Fig 3. The cost-effectiveness plane showed that the mean incremental costs per person with the capsaicin 8% patch were £22.50 and the mean incremental QALYs gained were 0.052. At a willingness-to-pay threshold of £20,000 per QALY gained, the probability of capsaicin 8% patch being cost-effective versus pregabalin was 97%.

## Discussion

Scottish treatment guidelines currently recommend pregabalin for adult patients with PNP who have failed first- or second-line pharmacological treatment [6]. Our economic model demonstrates that capsaicin 8% patch is a dominant (more effective with lower costs) treatment option in this patient population compared with pregabalin and therefore would be considered cost-effective from the perspective of the NHS and Personal and Social Services in Scotland. In the base-case analysis and the majority of sensitivity analyses, the capsaicin 8% patch treatment strategy remained dominant compared with pregabalin.

The robustness of the base-case findings were supported by extensive sensitivity analyses. Capsaicin 8% patch remained dominant in the scenario analyses which assumed equivalent efficacy of capsaicin 8% patch and pregabalin, no differences in time to response between treatments, no difference in discontinuation due to intolerable adverse events between treatments, no difference in pain response utilities between treatments, a combination of all of these assumptions, as well as a scenario which assumed that patients who received capsaicin 8% patch would experience a reduction in pain relief before re-treatment. Capsaicin 8% patch also remained dominant or cost-effective in the one-way sensitivity analysis. A structural sensitivity analysis showed that the capsaicin 8% patch was the dominant treatment strategy over all time horizons except for 1 year, when the ICER was £1,242 per QALY gained (i.e. highly cost-effective). The probabilistic sensitivity analysis showed that varying model inputs had limited impact on the results, and the probability that the capsaicin 8% patch was cost-effective compared with pregabalin at a willingness-to-pay threshold of £20,000 per QALY was 97%.

The model was most sensitive to variations in the time to re-treatment with the capsaicin 8% patch. Current labelling suggests that the capsaicin 8% patch may be reapplied every 90 days as warranted by the persistence or return of pain [7]. However, real-life data from the ASCEND non-interventional study, which was conducted across six European countries, suggest that retreatment occurs less frequently than this (mean 179 days) and is required by only 31% of patients [30]. We applied the re-treatment intervals from the ASCEND study in our model. When the time to re-treatment was reduced to the lower 95% confidence interval (117 days), the ICER increased to £7,951 per QALY, which is well below the £20,000 per QALY willingness-to-pay threshold most commonly adopted by the Scottish Medicines Consortium and NICE [12, 29]. Further, the threshold analysis showed that for treatment with the capsaicin 8% patch to exceed the £30,000 per QALY willingness-to-pay threshold either: time to re-treatment would need to be less than 60.5 days, considerably less than the 90-day interval suggested in the labelling [7]; or that the average number of patches per treatment would need to be greater than 4.10, more than the maximum of 4 patches per application specified by the label [7] and considerably more than the mean number of capsaicin patches used per treatment as seen in the ELEVATE study: 1.38 (standard deviation [SD] 1.08).

A key data source for our model was the ELEVATE study, a phase IV, randomized, non-inferiority, multicentre trial, which compared the efficacy and tolerability of capsaicin 8% patch with pregabalin in a broad population of patients with non-diabetic PNP (including post-herpetic neuralgia, peripheral nerve injury and painful peripheral polyneuropathy). ELEVATE demonstrated the non-inferiority of capsaicin 8% patch versus pregabalin in pain reduction (i.e. ≥30% decrease in average NPRS score from baseline to week 8) and also showed that the median time to pain relief was shorter with capsaicin 8% patch (7.5 versus 36 days) [11]. These data were included in the model and contributed to the better cost-effectiveness of capsaicin 8% patch compared with pregabalin. ELEVATE also showed that capsaicin 8% patch and pregabalin had different tolerability profiles, with local events being more common with capsaicin 8% patch, and dizziness, somnolence, headache and nausea being more common with pregabalin [14]. While adverse events were not considered in our cost-effectiveness model, the responder health-state utility values were taken from the ELEVATE study. The value for capsaicin 8% patch (0.23) was slightly higher than for pregabalin (0.20), indicating a better HRQoL, which was attributed to differences in the tolerability profiles of the two treatments. Modelling in this way removed the need to consider adverse events separately or use external estimates of the impact on HRQoL. If we had applied HRQoL decrements for adverse events reported in the literature (e.g. dizziness, ‒0.12 [31]; nausea, ‒0.065 [32]), and assumed that these decrements persisted during pregabalin treatment at the rate observed in the ELEVATE study (approximately 30%/day) [17], the total decrements would have been approximately 0.02 or 0.04 per year. These values are similar to the 0.03 utility difference applied in our model.

Two previously published systematic literature reviews and meta-analyses of eligible studies have indicated that the numbers needed to treat (versus active control) for the capsaicin 8% patch were higher than that for pregabalin: Finnerup et al reported NNTs of 10.6 (95% CI 7.4–19.0) versus 7.7 (95% CI 6.6–9.4), respectively, over 12 weeks [33]; Derry et al reported NNTs of 7.0 (95% CI 4.6–15.0) versus 5.4 (95% CI 3.9–9.2), respectively [34]. However, the data for the capsaicin 8% patch and pregabalin were analyzed independently in these studies. An assessment of relative efficacy between capsaicin 8% patch and pregabalin from these analyses does not the meet the criteria for an indirect treatment comparison or network meta-analysis [35]. Furthermore, the independent studies in the meta-analyses are likely subject to a degree of bias of heterogeneity in study design, PNP etiologies, comparators, etc, and therefore provides inadequate comparability for capsaicin 8% patch versus pregabalin. The results of ELEVATE were published after these published systematic literature reviews/meta-analyses were performed and therefore do not include ELEVATE. The ELEVATE study was open label due to the difficulty in selecting a blind control, but represents a randomized head-to-head study of two active comparators providing direct evidence to inform the comparison in this analysis.

To the best of our knowledge, there are only two other cost-effectiveness analyses involving the capsaicin 8% patch [36, 37]. In one analysis, capsaicin 8% patch was compared with nortriptyline (as a representative tricyclic antidepressant), lidocaine 5% patch, duloxetine, gabapentin and pregabalin in patients with post-herpetic neuralgia [36]. The Markov model had a time horizon of 1 year and was conducted from the perspective of a US managed care organisation. Mean ICERs for capsaicin 8% patch were below the $50,000 to $100,000 per QALY willingness-to-pay threshold generally applied in the US for all oral comparator agents (i.e. $59,919 versus tricyclic antidepressants; $43,908 versus duloxetine; $42,008 versus gabapentin; $40,241 versus pregabalin), except versus lidocaine 5% patch ($554,627) [36]. The model had some similarities to our own (i.e. responders defined using ≥30% pain improvement cut-off, patients assumed to remain on therapy or discontinue treatment due to adverse events), but included additional factors (i.e. dose titration, adverse events) not considered in our model. The high ICERs reported by Armstrong et al. [36] are likely to be attributable, in part, to the base-case assumptions that the capsaicin 8% patch was re-applied every 12 weeks and that patch application required 2 to 2.5 hours and was performed by a physician. In the other analysis, performed by the North East Treatment Advisory Group in the UK [37], the estimated annual cost of the capsaicin 8% patch was £2932, which assumed 2 patches per treatment, 4 patches per year (i.e. given at 90-day intervals) and that patches were given in a specialist pain clinic, and the estimated annual cost effectiveness was £21,000 for one patient to achieve ≥2 point reduction in pain score. These estimates are higher than in our analysis because of the different times for re-treatment (i.e. every 90 versus 179 days) and the high tariff for a pain specialist clinic.

Our model included several assumptions where no data were available to provide a firm estimate. Many of these assumptions were conservative and may have underestimated the benefits of the capsaicin 8% patch, e.g. all patients were assumed to be treated a second-time with capsaicin 8% patch, yet non-interventional study data suggest that only 31% of patients are re-treated [28]; the costs of managing adverse events were not included; adherence with pregabalin was assumed to be 100%, although it is likely to be less in clinical practice (~50%) [38]; and a grade 6 nurse applied the capsaicin 8% patch, whereas input from clinical experts suggested that the procedure may be performed by a less qualified nurse in clinical practice. As there is no dominant treatment pathway for the management of PNP in Scotland, assumptions were also necessary regarding the model structure, the most appropriate comparator and subsequent therapy. All of these assumptions were validated by two pain specialists who practice in Scotland. The findings from this model are therefore directly relevant to the healthcare environment in Scotland alone and may not be suitable for extension to other countries.

This economic analysis suggest that capsaicin 8% patch is a cost-effective treatment option compared with pregabalin for patients with PNP who have not tolerated or have not achieved adequate pain relief from conventional first- and second-line treatments from the perspective of the NHS and Personal and Social Services in Scotland.

## Supporting Information

S1 Table Model assumptions. (DOCX)

S1 Fig Utility scores assuming reduction in pain relief over time with capsaicin 8% patch. (EPS)

S2 Fig Modelling of utilities over time for treatment responders. (EPS)
