# Supplementary material for: Cost-Effectiveness of Capsaicin 8% Patch Compared with Pregabalin for the Treatment of Patients with Peripheral Neuropathic Pain in Scotland
Source: PLoS One. 2016 Mar 16;11(3):e0150973. doi: 10.1371/journal.pone.0150973 (PMC4794144; doi:10.1371/journal.pone.0150973)
Supplement: S1 Table — (DOCX) [file pone.0150973.s001.docx]

**S1 Table. Model assumptions.**

| - Patients who responded to treatment experienced a linear increase in utility from the baseline value for the median time to pain relief by the increment associated with pain relief. |
| --- |
| - Patients who had an initial response to any treatment continued to respond to that treatment for the period modelled. |
| - All patients treated with capsaicin 8% patch and who responded were retreated. |
| - Pain relief started to decrease with the capsaicin 8% patch after 8 weeks (scenario analysis only). |
| - Patients receiving last-line therapy responded immediately. |
| - Patients failing to respond to last-line therapy are assumed to continue to incur the cost of therapy regardless of response status. |
| - There are no additional costs incurred for the management of adverse events. |
| - Routine monitoring occurred at equal frequency with the two treatment strategies. |
| - A grade 6 nurse was required to apply capsaicin 8% patches. |
| - Pregabalin was given as a twice-daily dosing regimen. |
| - Adherence with pregabalin and last-line therapy was 100%. |
| - Intolerable adverse events led to a general practitioner visit and a pain specialist visit. |
| - Duloxetine was used as a proxy for estimating the cost of last-line therapy. |
| - One pair of nitrile gloves was required for the application of each capsaicin 8% patch. |
